# Comparison of two methods for prolong storage of decellularized mouse whole testis for tissue engineering application: An experimental study

**DOI:** 10.18502/ijrm.v19i4.9058

**Published:** 2021-04-22

**Authors:** Nasrin Majidi Gharenaz, Mansoureh Movahedin, Zohreh Mazaheri

**Affiliations:** ^1^Anatomical Sciences Department, Faculty of Medical Sciences, Tarbiat Modares University, Tehran, Iran.; ^2^Basic Medical Science Research Center, Histogenotech Company, Tehran, Iran.

**Keywords:** Cryopreservation, Testis, Scaffold, Mouse.

## Abstract

**Background:**

Biological scaffolds are derived by the decellularization of tissues or organs. Various biological scaffolds, such as scaffolds for the liver, lung, esophagus, dermis, and human testicles, have been produced. Their application in tissue engineering has created the need for cryopreservation processes to store these scaffolds.

**Objective:**

The aim was to compare the two methods for prolong storage testicular scaffolds.

**Materials and Methods:**

In this experimental study, 20 male NMRI mice (8 wk) were sacrificed and their testes were removed and treated with 0.5% sodium dodecyl sulfate followed by Triton X-100 0.5%. The efficiency of decellularization was determined by histology and DNA quantification. Testicular scaffolds were stored in phosphate-buffered saline solution at 4°C or cryopreserved by programmed slow freezing followed by storage in liquid nitrogen. Masson's trichrome staining, Alcian blue staining and immunohistochemistry, collagen assay, and glycosaminoglycan assay were done prior to and after six months of storage under each condition.

**Results:**

Hematoxylin-eosin staining showed no remnant cells after the completion of decellularization. DNA content analysis indicated that approximately 98% of the DNA was removed from the tissue (p = 0.02). Histological evaluation conﬁrmed the preservation of extracellular matrix components in the fresh and frozen-thawed scaffolds. Extracellular matrix components were decreased by 4°C-stored scaffolds. Cytotoxicity tests with mouse embryonic fibroblast showed that the scaffolds were biocompatible and did not have a harmful effect on the proliferation of mouse embryonic fibroblast cells.

**Conclusion:**

Our results demonstrated the superiority of the slow freezing method for prolong storage of testicular scaffolds.

## 1. Introduction

Tissue engineering produces constructs for replacing damaged tissues or organs and provides a useful alternative to conventional treatments (1). It is the application of decellularized tissues as three-dimensional scaffolds, cells, and growth factors for the establishment of functional tissue or organ for replacement. Decellularization removes cells of tissues using detergent while preserving the component of extracellular matrix (ECM) including proteins, glycosaminoglycan (GAG), and their biology. These scaffolds help the formation of new tissues by improving cell-cell interactions (2, 3). A perfect scaffold for tissue engineering should facilitate nutrient distribution, cell proliferation, and provide a platform similar to the structure of the tissue that needs to be replaced. Decellularized scaffolds are tissue-specific and non-immunogen in nature; thus, they are considered ideal scaffolds in tissue engineering (4). These scaffolds facilitate the re-engineering of many different tissues in clinical settings (5). Decellularized testicular scaffolds can be used for the development in vitro spermatogenesis, treatment of infertility with male factor, the study of the spermatogenesis, and genetic modification of the stem cells (6). Spermatogenesis is a multipart process; in this process, spermatogonial stem cells proliferate and differentiate to haploid sperm in an appropriate microenvironment and niche. It was started by migration of male germ line cells to the basement membrane of seminiferous tubules of testes and continues with proliferation and differentiation of them to sperm (7). Biological scaffolds, such as ECM, provide a suitable microenvironment for the homing and proliferation of spermatogonial stem cells. These scaffolds appear to act as an optimal bio-construct for medical needs. Despite the growing interest on the potential use of decellularized whole testes as three-dimensional scaffolds for the establishment in vitro spermatogenesis, optimal storage conditions are not well-defined.

The storage and quality of decellularized scaffolds are critical for the success in the re-cellularization of scaffolds and their in vivo transplantation (4). The preservation structure and composition of ECM components is important in facilitating cell-matrix interactions and retaining important structural and functional signals for promoting cell proliferation (8, 9). Improper preservation may significantly affect scaffold behavior. Lack of suitable protocol for prolonging the storage of decellularized scaffolds remains challenging. To date, no appropriate method for cryopreservation testicular scaffolds has been developed. In Thus, in this study, we prepared decellularized scaffolds from mouse whole testes and compared slow freezing method and storage at 4°C for prolong storage of testicular scaffolds.

## 2. Materials and Methods 

### Organ harvesting and decellularization protocol 

In this experimental study, 20 male NMRI mice (8 wk) were used for the production of testicular scaffolds. The animals were housed in controlled environmental conditions (12-hr light/dark cycles) with free access to food and water.

Mice were exposed to chloroform (Sigma, USA) and then sacrificed by cervical dislocation. After that, the capsules of testes were pierced using an insulin syringe 29 gauge. For the elimination of residual blood, testes were washed with phosphate-buffered saline (PBS; Invitrogen, Basel, Switzerland); later, the washed testes were decellularized at room temperature on an orbital shaker (50 RPM) with 0.5% (v/v) sodium dodecyl sulfate (SDS; Sigma, USA), diluted in distilled water for 18 hr + 0.5% (v/v) Triton X-100 (Sigma, USA) diluted in distilled water for 18 hr. Following the decellularization, the scaffolds were washed extensively with PBS for 24 hr, disinfected using 0.1% peracetic acid in 4% ethanol for 2 hr, and rinsed thrice with sterile PBS; each rinsing cycle was run for 4 hr (10).

### Storage protocol

#### Slow freezing 

The scaffolds were equilibrated in freezing media, including Dulbecco's Modiﬁed Eagle's Medium (DMEM; Thermo Fisher Scientific Inc.) and 10% dimethyl sulfoxide (DMSO; Sigma, USA). The scaffolds, along with 0.9 mL of the freezing medium, were packaged into cryovials at room temperature. The cryovials were then loaded into a programmable freezer (Kryo 360, UK) and subjected to a deﬁned freezing program. The freezing program consist of keeping the cryovials at 22°C for 10 min, cooling to 4°C at -1°C/min, keeping at 4°C for 5 min, cooling at 0.3°C/min from 4°C to -8°C, keeping at -8°C for 10 min, cooling at 0.5°C/min from -8°C to -50°C, followed by cooling at 10°C/min from -50°C to -90°C, and keeping for 10 min at -90°C. At -90°C, the cryovials were transferred into liquid nitrogen (LN) and stored until further analysis. For thawing, scaffolds were melted in a 37°C water bath (11).

#### Four degrees (4°C)

Scaffolds were placed in 15-ml falcon in PBS with penicillin/streptomycin and stored at 4°C.

### Scaffold analysis 

Testicular scaffolds (five in number) were analyzed after decellularization and compared to native testes. Scaffold characterization was conducted after six months of storage at 4°C or liquid nitrogen. A minimum of five samples from different animals were used for each test.

### Histological analysis

The samples were ﬁxed in formalin solution (10%) at room temperature for 24 hr, dehydrated in graded alcohol. They were cut into 5 µm-thick sections following embedding in paraffin. H&E (Sigma, USA) staining was performed on paraffin sections of the samples to examine the efﬁcacy of the decellularization method. For the qualitative evaluation of collagen and GAG retention after decellularization and storage, Alcian blue (Sigma, USA) and Masson's trichrome staining were done (12, 13).

### DNA content analysis 

Extraction of DNA was done using a QIAamp DNA Mini Kit (Qiagen, Germany). A minimum of five samples from different animals were used for each test. For measuring DNA content, a Nano Drop 2000 C UV-Vis spectrophotometer (Thermo Scientific, Venlo, Netherlands) were used.

### Immunohistochemistry

The retention of ECM proteins including laminin, collagen IV, and fibronectin following decellularization and storage was evaluated by immunohistochemistry.

For the antigen retrieval, the slides were heated in 10-mM citrate solution for 30 min until the temperature reached 95-100°C. Normal goat serum (Sigma, USA) was used for blockage unspeciﬁc antigen binding sites. The primary antibodies were anti-fibronectin (mouse monoclonal IgG, E-AB-22077, Elabscience Biotechnology Inc.), anti-collagen IV (mouse monoclonal IgG, E-AB-22150, Elabscience Biotechnology Inc.), and anti-laminin (rabbit polyclonal IgG, ab11575, Abcam). The secondary antibodies were Alexa Fluor 488 (goat anti-mouse IgG, A1100, Invitrogen) or Texas Red (Goat anti-rabbit IgG, ab6719, Abcam). Images were taken using an Olympus microscope (Olympus, Center Valley, PA).

### Collagen and GAG quantiﬁcation

Concentrations of collagen and GAGs in native testes, fresh and stored scaffolds were quantified using Sircol assay kit (Tebu-Bio, Belgium, S1000) and Blyscan assay kit (Tebu-Bio, Belgium, B1000), respectively, according to the manufacturer's instructions.

### Cytotoxicity assay 

The cytotoxicity of produced scaffolds was evaluated using 3-[4, 5-dimethyl (thiazol-2yl)-3,5diphenyl] tetrazolium bromide (MTT; Sigma, USA) test. The isolation of Mouse embryonic fibroblast (MEF) cells was done using Jozefczuk's protocol (14). The isolated cells were cultured at a density of 3 × 104 cells/well on 2 × 2 × 2 mm3 fragments of the scaffolds in DMEM with 10% fetal bovine serum (FBS; GIBCO, Germany) for 72 hr. The viability of cells was examined after 24 and 72 hr using the following protocol. Initially, the cells of each well were incubated with 200 µL medium containing 0.5 mg/mL MTT (Sigma, USA) for 4 hr at 37°C for formazan formation. Then, the medium was removed and DMSO was added for dissolving the formazan. A microplate reader (Beckman, Fullerton, CA) was used for measuring of optical density (OD) of the supernatants. A minimum of five samples from different animals was used for each test (15).

### Spermatogonial cell attachment

Cell attachment was evaluated by seeding spermatogonial cells on the frozen-thawed scaffolds. These cells were isolated by an enzymatic digestion process of mice testes, as described by Mirzapour and colleagues (16). Spermatogonial cells at density of 1 × 10${}^{5}$ per ml were seeded on scaffolds and cultured in DMEM medium with FBS for seven days. Following the seven days of incubation, the cell-seeded scaffolds were washed thrice with PBS to remove non-attached cells, and then they were ﬁxed, sectioned, and stained with H&E or 4',6-diamidino-2-phenylindole (DAPI).

### Ethical considerations

All animal procedures were conducted following the guidelines approved by the ethical committee of the Medical Sciences Faculty at the Tarbiat Modares University (Permission No. IR.TMU. REC.1394.269).

### Statistical analysis

Data were analyzed using the SPSS software (Statistical Package for the Social Sciences, version 16.0, Chicago, USA). One-way analysis of variance (ANOVA) and Tukey's post-hoc test was used for statistical analysis. All data are presented as mean ± standard deviations. The experiments were replicated five times. P ≤ 0.05 was considered as statistically significant.

## 3. Results

### Characterization of testicular scaffolds 

**Figure 1:**
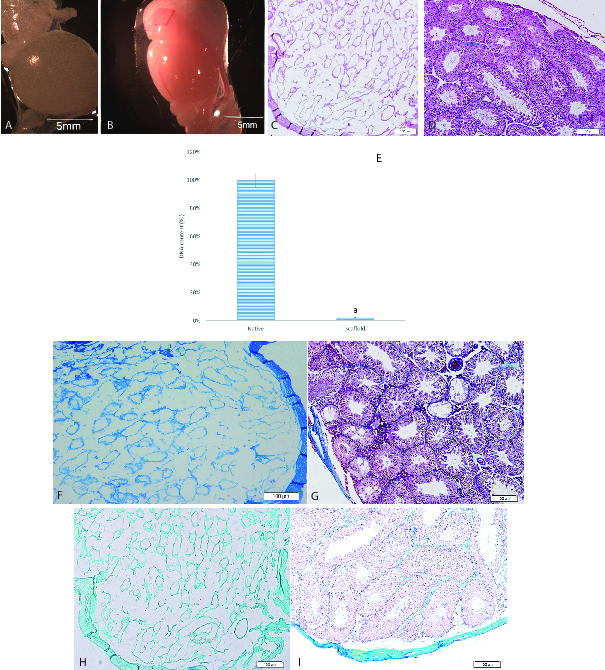
Characterization of testicular scaffolds. Macroscopic images showed that scaffolds were completely translucent (A) while native testes were opaque (B). Histological comparison of scaffolds (C) and native testes (D) by H&E staining exhibited the elimination of the cells. Original magnification 100x. DNA quantification confirmed the removal of 98% of the DNA from the tissue (E). a indicated significant difference with native testis (p < 0.05). Masson's trichrome staining showed collagen preservation in scaffolds (F) and native testes (G). Alcian blue staining confirmed GAGs retention in scaffolds (H) and native testes (I). Original magnification 100×.

Isolated mouse testes were decellularized using detergents. Our decellularization protocol effectively removed all cellular material. The removal of cellular components was reﬂected by the color change in the testes during the decellularization process. Macroscopic imaging showed that the scaffolds are translucent while native testes are opaque (Figures 1A and 1B). H&E staining showed that cells were removed after the completion of decellularization and empty seminiferous tubules were distinguishable in scaffolds. Also, the scaffolds maintain the architecture of ECM and basal lamina compared to the native testis (Figures 1C and 1D). The efficacy of the decellularization process was evaluated by DNA quantiﬁcation. Spectrophotometric analysis indicated that approximately 98% of the DNA was removed from the tissue, conﬁrming the efficiency of the decellularization method (p = 0.02) (Figure 1E). The specific staining using Masson's trichrome staining exhibited the preservation of blue-stained collagen fibers in the scaffolds. Though no red-stained areas, indicating cell fragments, were detected in the scaffolds (Figures 1F and 1G). Alcian blue staining confirmed the preservation of GAGs in scaffolds compared to the native testis (Figures 1H and 1I).

**Figure 2:**
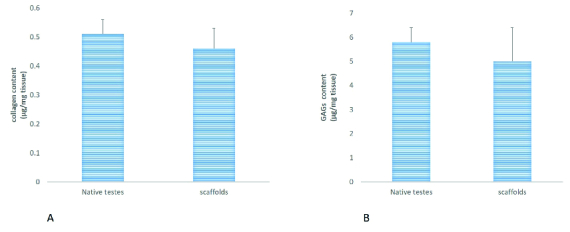
Quantification of collagen and GAGs content in native and scaffolds. Collagen in native and scaffolds was quantified using the Sircol assay (A). Quantification of GAGs in native and scaffolds using the Blyscan assay (B). Results are presented as Means ± SD (p < 0.05).

Quantification of total collagen and GAGs content showed that there was no significant reduction in collagen and GAGs level in scaffolds relative to native testes (Figures 2A and 2B).

**Figure 3:**
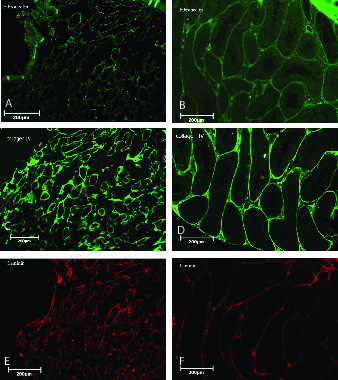
Immunohistochemical and ultrastructural analyses of testicular scaffolds and native testes. Representative images of fibronectin expression in scaffolds (A) and native testis (B), collagen IV expression in scaffolds (C) and native testis (D), and laminin expression in scaffolds (E) and native testis (F). Original magnification 100×.

Immunohistochemistry confirmed the preservation of ECM proteins including ﬁbronectin (Figure 3A), collagen IV (Figure 3B), and laminin (Figure 3C) in the scaffolds. Native testes were stained as control (Figures 3D, 3E, and 3F).

**Figure 4:**
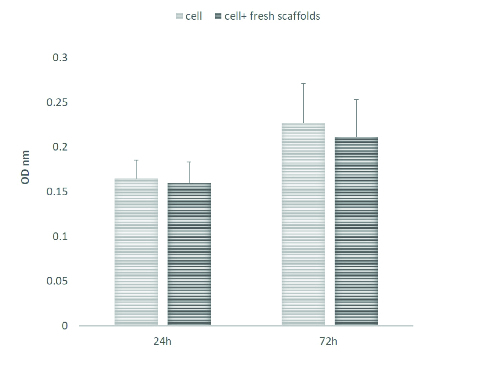
Evaluation of scaffold cytocompatibility. The result of the MTT test did not show any significant difference in the optical density (OD) values, meaning that the cells proliferated at a rate similar to that of the controls.

Evaluation of the cytotoxicity of the scaffold using the MTT test revealed that decellularized scaffolds had no major effect on the proliferation of MEF cells after 24 and 72 hr of cultivation (Figure 4).

### Evaluation of frozen-thawed scaffolds

**Figure 5:**
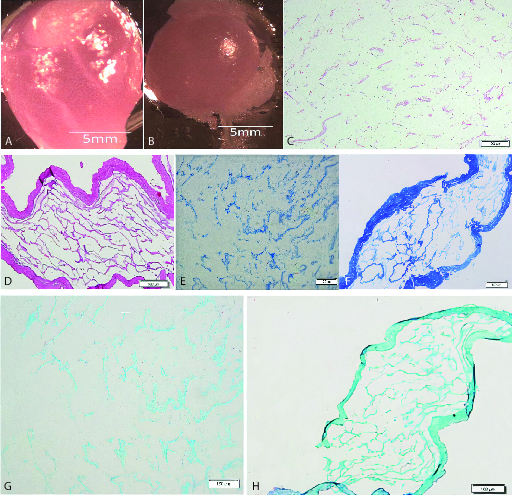
Characterization of testicular scaffolds after storage. Representative image of macroscopic appearance (A&B), H&E staining (C&D), Masson's trichrome staining (E&F), Alcian blue staining (G&H) of scaffolds after storage at 4°C or cryopreservation by slow freezing method, respectively. The images confirmed the superiority of the slow freezing method in the preservation of the ECM component. Original magnification 100×.

From a macroscopic viewpoint, frozen-thawed scaffolds exhibited a pink color owing to the immersion in a medium containing phenol red. Except for the color, the frozen-thawed scaffolds were similar to fresh scaffolds, while 4°C scaffolds stored for six months showed degradation and loss of consistency (Figures 5A and 5B). Additionally, the H&E staining demonstrated that the frozen-thawed scaffolds preserved the basal lamina of the seminiferous tubule. while the 4°C-stored scaffolds showed the destruction of seminiferous tubule (Figures 5C and 5D). Moreover, Masson's trichrome staining of frozen-thawed scaffolds confirmed the good maintenance of collagen fibers but 4°C-stored scaffolds showed a decrease in collagen fiber (Figures 5E and 5F). Alcian blue staining highlighted changes in the GAGs present in the ECM. The GAGs were well-preserved in frozen-thawed scaffolds while it was only partially preserved in 4°C scaffolds (Figures 5G and 5H).

**Figure 6:**
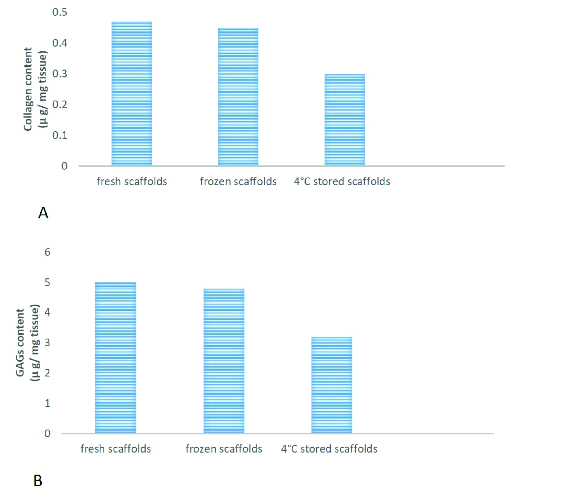
Quantification of collagen and GAGs content in scaffolds after storage. Collagen in 4°C-stored and frozen-thawed scaffolds were quantified using the Sircol assay (A). Quantification of GAGs in 4°C-stored and frozen-thawed scaffolds using the Blyscan assay (B). Results are presented as Mean ± SD (p < 0.05).

A deeper analysis using quantitative assays showed that collagen and GAGs levels were similar in the frozen-thawed scaffolds and fresh scaffolds while they were decreased in the 4°C-stored scaffolds (Figures 6A and 6B).

**Figure 7:**
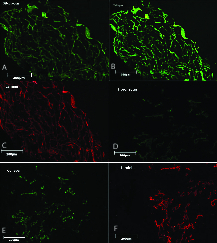
Immunohistochemical analyses of frozen-thawed scaffolds. Representative images of fibronectin, collagen IV, and laminin expression in frozen-thawed (A-C) and 4°C-stored scaffolds (D-F). Original magnification 100×.

In addition, the immunohistochemistry analysis demonstrated that the expression pattern of ECM proteins including ﬁbronectin, collagen IV, and laminin in frozen-thawed scaffolds was similar to that in the fresh scaffolds (Figures 7A-7C). On the other hand, the 4°C-stored scaffolds showed poor expression of ECM proteins (Figures 7D-AF).

**Figure 8:**
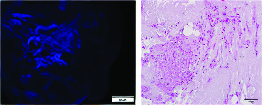
Evaluation of cell attachment to the frozen-thawed scaffold. Spermatogonial cells were seeded directly on the frozen-thawed scaffolds to study cell attachment. Cell culture was carried out for up to seven days. Spermatogonial-cell attachment and inﬁltration as seen after seven days by DAPI staining (A) and H&E staining (B). Original magnification 200×.

Further, spermatogonial cells were indirectly exposed to frozen-thawed scaffolds to test whether the scaffolds were biocompatible and to analyze cell attachment. Our results show that the frozen-thawed scaffolds exerted no cytotoxicity on spermatogonial cells (Figures 8A and 8B).

## 4. Discussion

The use of decellularized tissues in clinics is increasing, but one of the major limitations for their clinical application is the lack of a standard protocol for the long-term preservation of these scaffolds. Recently, the application of decellularized testicular scaffold for spermatogenesis induction has increased (17-19).

Despite growing interest on the potential use of decellularized whole testes as three-dimensional scaffolds for establishment in vitro spermatogenesis, optimal storage conditions are not well-defined. In this study, two methods were used for the long-term storage of decellularized testicular scaffolds. Initially, we showed that the use of Triton X-100 and SDS is a suitable method for the decellularization of whole testes. Our results were consistent with previous studies on the decellularization of different types of tissues including tendon-bone, small-diameter blood vessels, and pericardium using SDS + Triton X-100 detergent (13, 20-22). Then, two usual storage methods including slow freezing and storage at 4°C were compared. One of the frequently used methods for storing tissues and cells is slow freezing with DMSO. Slow freezing with DMSO has been used successfully for heart-valve storage and ECM integrity was preserved (23). Another ordinary storage method is storage in the refrigerator. Biologic scaffolds such as bone, cartilage, and skin can be stored for prolonged periods of time prior to use (24, 25). Decellularised esophagi were stored in LN for six month (26). On the other hand, Bonenfant and colleagues reported that decellularized lungs could be stored beyond three months in refrigerator, but they lose their native architecture after six months (25). However, it is unclear which one of these storage protocols will be appropriate for decellularized whole testis. Thus, testicular scaffolds were frozen using DMSO or maintained at 4°C. From a macroscopic viewpoint, important differences were not seen in the frozen-thawed scaffolds compared to the 4°C-stored scaffolds. On the contrary, storage in 4°C led to changes in the scaffold with loss of consistency. The preservation of scaffolds structure and important proteins of ECM are necessary for cell attachment and proliferation following recellularization. Histological evaluation and quantification assays for ECM components showed collagen and GAGs preservation only in frozen-thawed scaffolds. Immunohistochemistry analysis confirmed the good expression of ECM proteins only in frozen-thawed scaffolds.

All these data demonstrated slow freezing is better than storage at 4°C for slow freezing for the long-term storage of testicular scaffolds. The efficacy of the slow freezing method for cell freezing is confirmed but it is not widely used for scaffolds storage and its effectiveness in decellularized testicular scaffolds storage was not evaluated. Several studies reported cell cryopreservation using DMSO and slow freezing in other tissues. Schenke-Layland and colleagues evaluated the ECM structures of heart-valve leaflets following cryopreservation. They reported the amounts of desmosine, elastin, and collagen were similar in fresh and cryopreserved samples (23). Brockbank and colleagues declared that cryopreservation porcine heart valves using slow freezing or vitrification has had comparable results even though artery tissues were significantly less viable in the vitrification method (27). In these studies, the effects of slow freezing on decellularized heart valves were not evaluated (23, 27). The effects of freezing on the structure of decellularized tissues might be different. Gallo and colleagues evaluated the effect of cryopreservation on decellularized aortic valves after implantation. Using echocardiography, they reported that there were no signs of stenosis, dilatation, and macroscopic calcification in both groups. Decellularized scaffolds could be repopulated with the recipient's fibroblasts, myofibroblasts, and smooth muscle cells; however, some destruction could be observed in several areas, which suggests a need for further improvements in the cryopreservation protocol (28). There is limited research in developing and evaluating effective protocols for the prolonged storage of scaffolds. We believe that the programmed slow freezing method proposed in this study, due to its inbuilt mechanism of cryoprotection, will allow the optimal preservation of decellularized testicular and other types of scaffolds as well.

## 5. Conclusion

Slow freezing mice testicular tissues did not have a harmful effect on colony formation rate of testicular cells. Our results showed that treating the mice whole testes with Triton X-100 and SDS efficiently removed the cells from the testes, so it is an appropriate protocol for the decellularization of whole testes. Testicular scaffolds could be cryopreserved by slow freezing. Our results demonstrated the superiority of the slow freezing method for prolong storage of testicular scaffolds.

##  Conflict of Interest

The authors declare no conﬂict of interest.
